# A retrospective analysis of amputation rates in diabetic patients: can lower extremity amputations be further prevented?

**DOI:** 10.1186/1475-2840-11-18

**Published:** 2012-03-02

**Authors:** Alexandra Alvarsson, Buster Sandgren, Carl Wendel, Michael Alvarsson, Kerstin Brismar

**Affiliations:** 1 Rolf Luft Centre for Diabetes Research, Department of Molecular Medicine and Surgery, Karolinska University Hospital, Karolinska Institutet, Stockholm, Sweden; 2 Department of Orthopedics, Karolinska University Hospital, Stockholm, Sweden; 3 Department of Trauma & Orthopedics, Danderyd University Hospital, Stockholm, Sweden; 4 Center for Molecular Medicine, L8:01, Karolinska University Hospital, SE 171 76 Stockholm, Sweden

**Keywords:** Lower extremity amputations, Diabetic foot, Foot ulcer, Diabetic complications

## Abstract

**Background:**

Lower extremity amputations are costly and debilitating complications in patients with diabetes mellitus (DM). Our aim was to investigate changes in the amputation rate in patients with DM at the Karolinska University Hospital in Solna (KS) following the introduction of consensus guidelines for treatment and prevention of diabetic foot complications, and to identify risk groups of lower extremity amputations that should be targeted for preventive treatment.

**Methods:**

150 diabetic and 191 nondiabetic patients were amputated at KS between 2000 and 2006; of these 102 diabetic and 99 nondiabetic patients belonged to the catchment area of KS. 21 diabetic patients who belonged to KS catchment area were amputated at Danderyd University Hospital. All patients' case reports were searched for diagnoses of diabetes, vascular disorders, kidney disorders, and ulcer infections of the foot.

**Results:**

There was a 60% reduction in the rate of amputations performed above the ankle in patients with DM during the study period. Patients with DM who underwent amputations were more commonly affected by foot infections and kidney disorders compared to the nondiabetic control group. Women with DM were 10 years older than the men when amputated, whereas men with DM underwent more multiple amputations and had more foot infections compared to the women. 88% of all diabetes-related amputations were preceded by foot ulcers. Only 30% of the patients had been referred to the multidisciplinary foot team prior to the decision of amputation.

**Conclusions:**

These findings indicate a reduced rate of major amputations in diabetic patients, which suggests an implementation of the consensus guidelines of foot care. We also propose further reduced amputation rates if patients with an increased risk of future amputation (i.e. male sex, kidney disease) are identified and offered preventive treatment early.

## Introduction

Foot ulcers are frequent and costly complications of diabetes [1], and the most common risk factor of lower extremity amputations (LEA) in diabetic patients [2,3]. Although debated, the rate of LEA has been considered an indicator of the quality of diabetic foot care [4]. Common diabetic complications such as peripheral neuropathy and peripheral vascular disease contribute to the formation of foot ulcers, the latter by causing ischemia, gangrene and impaired wound healing [5,6]. A common occurrence of ulcer infections in patients with foot ulcers is a contributing risk factor for LEA [7], along with renal disorders [8].

The St. Vincent Declaration was published in 1989 to set 5-year targets for the quality level of diabetes care in Europe [9]. In 1998 the Swedish Medical Research Council published "Consensus statement. Foot problems of Diabetics", and in 2000 the International Working Group on the Diabetic Foot published the "International Consensus on the Diabetic Foot and Practical Guidelines on the Management and the Prevention of the Diabetic Foot", which contained guidelines for quality care and prevention of the diabetic foot, stressing the importance of a multidisciplinary approach in order to ensure the most effective treatment [10,11]. Following these important publications several multidisciplinary studies have shown a decreased LEA rate following the introduction of national prevention programs and the establishment of multidisciplinary treatment teams for diabetic foot ulcers [12-14]. Consistently, multidisciplinary treatment teams headed by diabetologists have been applied at the Department of Endocrinology, Metabolism and Diabetes (DEMD) at the Karolinska University Hospital in Solna (KS) since the early 1990s. The primary care centers and hospitals in the region have repeatedly been offered theoretical educational courses on the preventive care of the diabetic foot, as well as in the treatment of diabetic foot complications. The aim of this study was to investigate clinical characteristics of diabetic patients amputated at KS, and to evaluate the impact of an implementation of the national and international guidelines on the diabetic foot by investigating the amputation rate. Since all personnel involved in the treatment of the diabetic foot at KS have to undergo education, including lectures and practical training, we decided to address the possible impact by investigating the treatment outcome, rather than by using surveys or interviews. Our hypothesis was that an awareness of the consensus guidelines will increase the quality of health care among all healthcare units and specialists in the multidisciplinary teams. Thus, prevention and treatment of diabetic foot ulcers will be more efficient, ultimately resulting in a reduced amputation rate in patients with diabetes. In this study, we report a reduction in the rate of amputations performed above the ankle in patients with diabetes treated at KS, during a period that followed the implementation of the consensus guidelines. We also identify risk groups that need extra attention in order to make it possible to reduce the amputation rates further.

## Subjects, materials and methods

**Figure 1:**
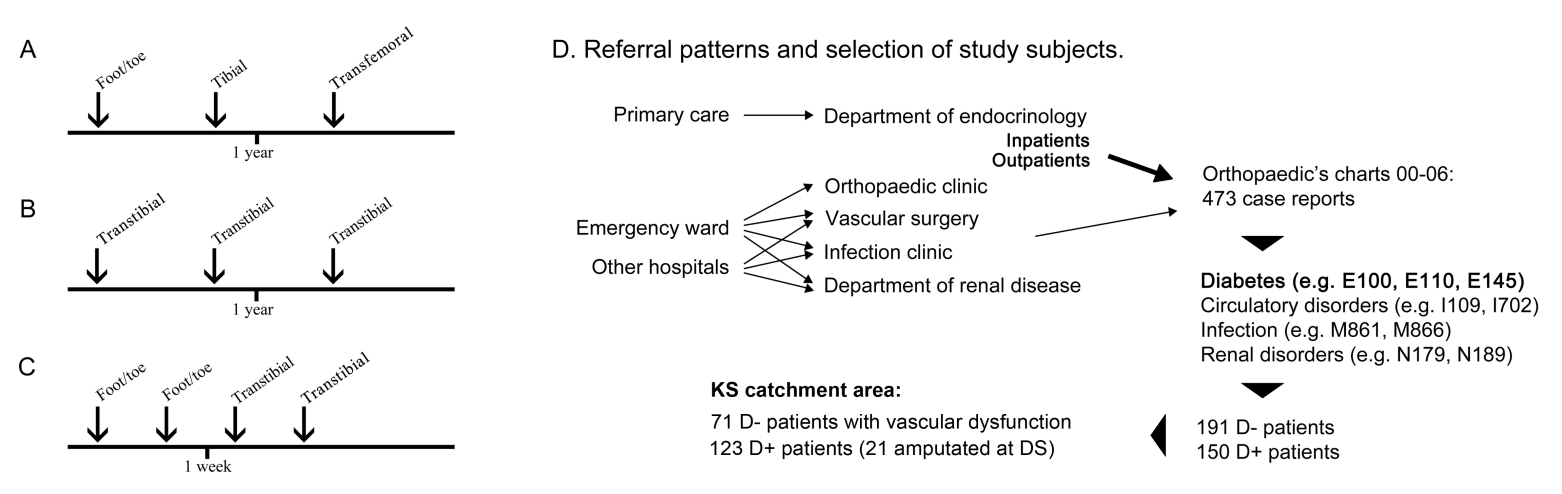
**Classification of amputations and selection of patients.** A, B and C. The arrows indicate events of amputations. D. Referral patterns and selection of study subjects.

The study was carried out at KS between January 2000 and December 2006. The catchment area of KS included 300 000 inhabitants and approximately over 10 000 subjects with diabetes during the study period, of which the majority were treated in the primary care. All patients who underwent orthopaedic procedures of the lower extremities at KS during the period (n = 473) were studied. The diagnoses were confirmed by searching through the listed patients' case reports for the event of an amputation preceded by a known history of diabetes mellitus. Patients who were diagnosed with diabetes mellitus at the time of amputation were defined as diabetic. 150 amputated patients with diabetes were found. 191 amputated patients without a known history of diabetes were defined as nondiabetic. When no or too little data could be retrieved from the patient's case report the patient was excluded. This was done in five cases, including the nondiabetic control group. Data from year 2000 was not complete due to a change in electronic case report systems at that time, thus, this year had to be excluded from all analyses across time to minimize the risk of underreporting. However, data collected from patients amputated year 2000 was used for group analyses in order to increase statistical power. The case reports of the 150 diabetic patients were searched for diagnoses of kidney disorders, vascular disorders and ulcer infections of the foot that were present before an amputation. Kidney disorders were defined as acute renal failure (ICD-10 code N17), chronic kidney disease (N18), known diabetes nephropathy (E10.2, E11.2, E14.2), serum creatinine levels > 100 μmol/l or kidney transplants. Infections were defined as positive culture of MRSA, osteomyelitis (M86), osteitis or infected wounds of the foot or residual limb (e.g. L02.4, L98.4, T86.4, T87.4, T81.4). Peripheral vascular disorders (PVD) were defined as atherosclerosis (I70), ischemia (I20), unspecified peripheral vascular disease (I73.9), arterial embolism and thrombosis (I74), diabetic circulatory disorders (E10.5, E11.5, E14.5), cerebrovascular diseases (I60-I69), diseases of veins (e.g. I80-I82, I87), or ischemia of the limb. Circulatory disorders were defined as PVD and cardiovascular disorders (CVD), the latter including hypertensive diseases (I10-I15), ischaemic heart diseases (I20-I25) and other forms of heart diseases (I30-I52). In addition, the 150 diabetic patients' case reports from the orthopaedic clinic were checked for the presence of foot ulcers at the event of amputation. All amputations performed below the ankle were defined as minor amputations, whereas amputations above the ankle were defined as major. No Syme's amputations or any other talocrural level amputations were performed. In cases where patients underwent multiple amputations during the study period, the amputation that was deemed to have the largest impact on the patient's quality of life was used for the analysis (Figure 1A). When patients underwent several amputations on the same level during an extended period of time, the first recorded amputation was used (Figure 1B). Repeated amputations (re-amputations) performed within the same period of hospital care, due to poor healing or infection, were classified as the last reported amputation within this period (Figure 1C). Re-amputations and subsequent amputations of a contralateral limb (double amputations) were analyzed separately.

The postal codes of the residential areas of the patients were retrieved from registers delivered from the Department of Orthopaedics, or from the patients' case reports, and were used to determine to which catchment area each patient belonged. The personal identification number, a unique 10 digit number that each resident in Sweden get at the time of birth or following immigration, was used for gender identification. Patient registers with personal identification numbers from the foot care policlinic were compared with patient registers from the orthopaedic's charts in order to identify patients who had been treated at the foot care clinic at the DEMD of KS during the time period. 21 patients belonging to KS catchment area were referred to Danderyd University Hospital (DS) for foot care and amputation during the investigated time period. This is the only diabetic patient group from KS catchment area known to have been treated at a hospital other than KS. Patients who underwent amputations at DS were identified using patient registers from the orthopaedic clinic at DS. Figure 1D shows the departments from which the diabetic patients were referred for amputation, the most common being the DEMD and the Department of Vascular Surgery. Patients from the DEMD were mostly referred for amputation due to complications of foot ulcers, while the majority of the patients from the Department of Vascular Surgery were referred due to inaccessibility for vascular intervention, failed reconstructions, or complications of vascular procedures.

The multidisciplinary foot team consisted of a diabetologist, a vascular surgeon, a specialist in infectious diseases, a chiropodist and an orthopaedic surgeon, who all were educated in the consensus guidelines for foot care. At the multidisciplinary foot clinic, patients were treated for hyperglycemia, hypertension and hyperlipidemia, and were given the opportunity to attend programs to quit smoking. Vascular surgeons performed percutaneous transluminal angioplasty or bypass surgery and, when necessary, a specialist in infectious diseases decided the antibiotic treatment, whereas orthopaedic surgeons decided on surgical revision and/or off-loading treatment. Diabetologists were responsible for metabolic control and, when necessary, the treatment of heart failure and kidney failure.

The diabetic and nondiabetic populations in KS catchment area were estimated based on the finding that 3.5% of the population between 40 and 70 years of age was diabetic in the geographically defined region Sundbyberg [15]. The total population numbers of each region in KS catchment area were retrieved from Statistics Sweden [16], using the age range 40-70 years. The diabetic population was estimated to constitute 3.5% of the total population. The nondiabetic population was calculated by subtracting the estimated number of diabetic patients from the total number of inhabitants in each region.

### Statistical analyses

STATISTICA 8 (StatSoft) and GraphPad Prism version 4.03 (GraphPad Software Inc.) were used for performing all statistical analyses. An unpaired Student's *t*-test was used to compare the mean age in female and male patients. Fisher's exact test was used to test for gender differences in the number of amputations, in the incidence or prevalence of additional diagnoses and foot ulcers, and in the referral for foot treatment. A one-way ANOVA analysis followed by a Bonferroni post hoc test was used to compare several groups of patients. Chi-square test for trend was used to study linear trends across time. A *p*-value < 0.05 was considered as significant.

## Results

### Patients from all catchment areas

The total number of patients amputated at KS between 2000 and 2006 was 341, of which 155 (45.5%) were females and 186 (54.5%) were males. The number of nondiabetic patients was 191, of which 98 (51%) were females and 93 (49%) were males. The number of diabetic patients amputated at KS between 2000 and 2006 was 150, of whom 57 (38%) were females and 93 (62%) were males. During the period of investigation at least ten different surgeons were performing amputations at KS. More than half of the amputations at KS were done by two surgeons with long experience (> 10 years). All surgeons had at least two years of orthopaedic specialist training with the same senior consultant as tutor before the amputations were performed.

### Patients from the catchment area of KS

Two hundred and one out of the 341 patients who were amputated at KS during the study period belonged to KS catchment area, 99 nondiabetic were amputated, of which 54 (54.5%) were females and 45 (45.5%) were males. In the nondiabetic group, 28 patients did not suffer from any vascular disorders but were mainly amputated due to neoplasms or physical trauma, whereas 71 nondiabetic patients suffered from vascular dysfunctions and were used as the nondiabetic control group. 102 of the 201 patients suffered from diabetes mellitus, 37 (36.5%) were females and 65 (63.5%) were males. Fisher's exact test revealed a significant difference in diabetes status and gender (*p *< 0.05), indicating that the gender distribution was skewed towards male gender in diabetic patients. The number of patients from KS catchment area amputated each year is displayed in Table 1, and the clinical characteristics are summarized in Tables 2 and 3. 21 diabetic patients were referred for amputations at DS during the study period, eight (38%) females and thirteen (62%) males. These patients underwent seventeen major and four minor amputations. Only age, gender and amputation status could be retrieved from these 21 patients. In order to increase statistical power, these patients were included when calculating the amputation rates.

**Table 1:** The nondiabetic patients with vascular dysfunction from KS catchment area amputated at KS, and diabetic patients from KS catchment area amputated at KS or DS (females/males)

Year	Number of patients		Transfemoral		Transtibial		Major		Minor	
	
	D-	D+	D-	D+	D-	D+	D-	D+	D-	D+
**2000**	**6** (3/3)	**17** (7/10)	2	5	4	9	6	14	0	3

**2001**	**10** (3/7)	**22** (6/16)	4	4	4	13	8	17	2	5

**2002**	**12** (8/4)	**20** (6/14)	5	3	6	15	11	18	1	2

**2003**	**4** (2/2)	**14** (7/7)	0	0	4	9	4	9	0	5

**2004**	**11** (4/7)	**24** (7/17)	2	2	7	9	9	11	2	13

**2005**	**11** (6/5)	**16** (9/7)	4	4	7	8	11	12	0	4

**2006**	**17** (13/4)	**10** (3/7)	2	2	9	5	11	7	6	3

**Total**	**71** (39/32)	**123** (45/78)	**19**	**20**	**41**	**68**	**60**	**88**	**11**	**35**

D-: patients without diabetes mellitus from the catchment area of KS amputated at KS due to vascular dysfunction; D+: patients with diabetes mellitus from the catchment area of KS amputated at KS or DS; major: transfemoral and transtibial amputations; minor: amputations of foot or toe. Re- and double amputations are not shown.

**Table 2:** Clinical characteristics of all amputated patients from the catchment area of KS

	D-	D+	
Number of patients	71	123	
Mean age at first amputation	81	75	*p *< 0.01
Median age at first amputation	83	78	

Number of amputations	77	166	
Number of multiple amputations	6 (7.7%)	43 (26%)	*p *< 0.001
Amputations per patient	1.08	1.35	

Patients with kidney disorders	3 (4%)	33 (32%)*	*p *< 0.0001
Patients with foot or limb infections	1 (14%)	17 (17%)*	*p *< 0.0001
Patients with PVD	71 (100%)	88 (86%)*	*p *< 0.001
Patients with PVD or CVD	71 (100%)	97 (95%)*	*p *= 0.08

Percentages in parenthesis indicate re-amputations per amputation, and diagnoses per patient, respectively. Multiple amputations: re- and double amputations.

*n = 102 in the D + group.

**Table 3:** Clinical characteristics of amputated diabetic patients from the catchment area of KS

	Females	Males	
Number of patients	45 (36.5%)	78 (63.5%)	
Mean age at first amputation	81.5	71.5	*p *< 0.001
Median age at first amputation	84	72	

Mean age in Swedish diabetic patients†	63.9	61.8	

Number of amputations	52	114	
Number of minor amputations	12 (23%)	47 (41%)	*p *< 0.05
Number of multiple amputations	7 (12%)	36 (34%)	*p *< 0.01
Amputations per patient	1.16	1.46	

Patients with kidney disorders*	8	25	*p *= 0.08
Patients with foot or limb infections*	2	15	*p *< 0.05
Patients with PVD*	35	53	*p *= 0.08
Patients with PVD or CVD*	37	60	

Patients affected by foot ulcers*	33 (89%)	57 (88%)	
Patients visiting the foot team*	7 (19%)	24 (37%)	*p *= 0.07

Percentages in parenthesis indicate minor amputations per amputation, re-amputations per amputation, and diagnoses per patient, respectively. Multiple amputations: re- and double amputations.

†NDR: The Swedish National Diabetes register, https://www.ndr.nu/NDR2/Default.aspx

*n = 102

### Amputation rates

**Figure 2:**
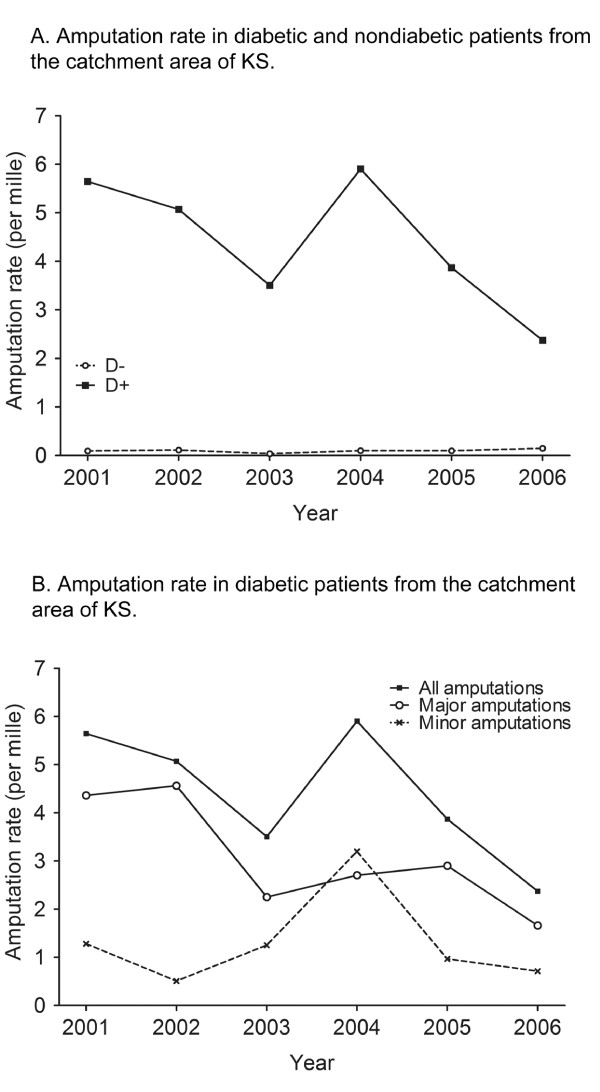
**Amputation rates across time.** A. Amputation rate in nondiabetic and diabetic patients from the catchment area of KS. B. Amputation rate in diabetic patients from the catchment area of KS.

In the 71 nondiabetic patients the amputation rate (the number of nondiabetic amputated patients divided by the nondiabetic population in KS catchment area) was 0.09 per mille in 2001 and 0.15 per mille in 2006 (see Figure 2A). A chi-square test for the trend did not reveal any significant linear trend in the total number of amputations performed throughout the study period. The rate of major amputations remained unchanged during the study period (*p *= 0.5), whereas there was a linear trend in the rate of minor amputations, which increased from 0.009 per mille to 0.05 per mille (*p *= 0.02) (data not shown). Six multiple amputations were performed in this group, giving 1.07 amputations per patient (see Table 2).

In the 123 diabetic patients the amputation rate changed from 5.60 per mille in 2001 to 2.4 per mille in 2006, with a tendency of a linear trend (*p *= 0.06) (Figure 2A and 2B). The rate of major amputations was 4.4 per mille in 2001 and 1.7 per mille in 2006 (*p *< 0.05), while the rate of minor amputations was 1.3 per mille in 2001 and 0.7 per mille in 2006 (Figure 2B) (*p *= 0.2). 43 multiple (re- or double) amputations were performed in this group, giving 1.35 amputations per patient, which was significantly more than in the nondiabetic group (*p *< 0.001). Male patients underwent a higher percentage of minor amputations compared to female patients (41% *vs*. 23%, *p *< 0.05). In line with this, male patients also underwent more multiple amputations (1.16 *vs*. 1.46 amp. per patient, *p *< 0.01) (see Table 3). Amputation rates in nondiabetic and diabetic patients from the catchment area of KS are displayed in table 4.

**Table 4:** Amputation rates in nondiabetic and diabetic patients from KS catchment area

Year	Inhabitants (40-70 years)	Nondiabetic			Diabetic		
	
		Population	Amputated (f/m)	Rate (per mille)	Population	Amputated (f/m)	Rate (per mille)
**2000**	110 082	106 229	**6** (3/3)	**0.056**	3853	**17** (7/10)	**4.4**

**2001**	111 356	107 459	**10** (3/7)	**0.093**	3897	**22** (6/16)	**5.6**

**2002**	112 728	108 783	**12** (8/4)	**0.11**	3945	**20** (6/14)	**5.1**

**2003**	114 176	110 180	**4** (2/2)	**0.036**	3996	**14** (7/7)	**3.5**

**2004**	116 211	112 144	**11** (4/7)	**0.098**	4067	**24** (7/17)	**5.9**

**2005**	118 200	114 063	**11** (6/5)	**0.096**	4137	**16** (9/7)	**3.9**

**2006**	120 452	116 236	**17** (13/4)	**0.146**	4216	**10** (3/7)	**2.4**

**Total**	n/a	n/a	**71** (39/32)	n/a	n/a	**123** (45/78)	n/a

### Age distribution

**Figure 3:**
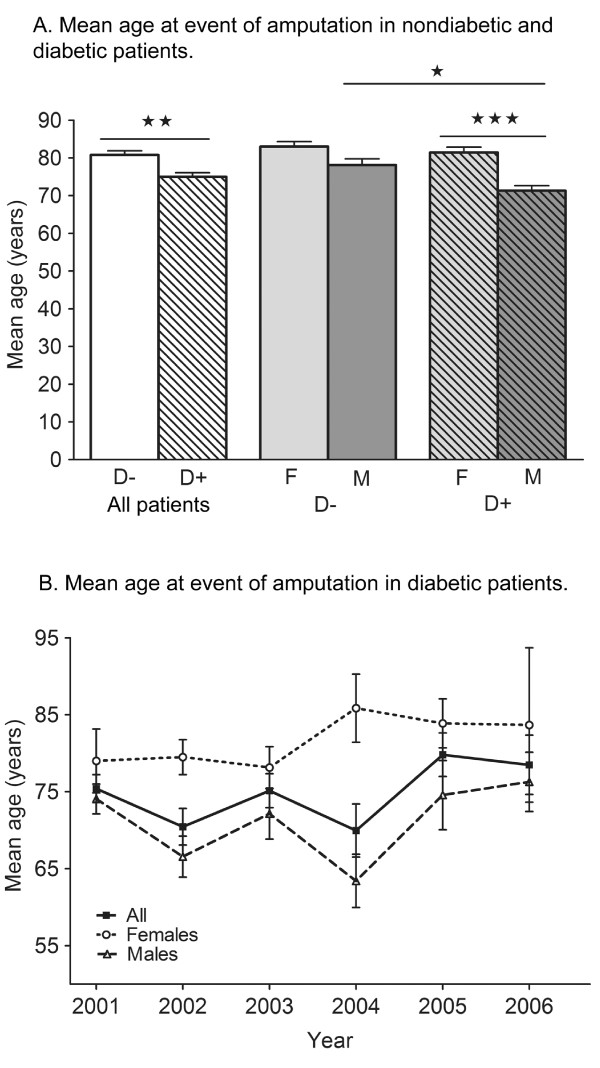
**Age at event of amputation.** A. Mean age at event of amputation in nondiabetic and diabetic patients. One-way ANOVA: p < 0.05, Bonferroni post hoc analysis: **p < 0.01 D- all patients *vs*. D + all patients, *** *p *< 0.001 D + female *vs*. D + male, *p < 0.05 D- male *vs*. D + male. Bars represent means + S.E.M. B. Mean age at event of amputation in diabetic patients. Error bars represent S.E.M.

In nondiabetic patients there was no age difference between the sexes (Figure 3A). The mean age at the event of amputation was 81 years (median: 83, range: 57-96) in the whole group, 83 years (median: 86, range: 71-96) in female patients and 78 years (median: 80.5, range: 57-95) in male patients.

The subjects in the diabetic group were significantly younger when undergoing amputations, compared to the nondiabetic group, as revealed by a one-way ANOVA followed by a Bonferroni post hoc test (*p *< 0.01) (Figure 3A). In the diabetic group the mean age was 81.5 years (median: 84) in female patients and 71.5 years (median: 72) in male patients at the event of amputation (see Figure 3A). The female patients were significantly older compared to the male patients (*p *< 0.001) when amputated. Diabetic men were significantly younger compared to nondiabetic men (*p *< 0.05), whereas there was no difference between nondiabetic and diabetic women. Figure 3B shows the annual age distribution in diabetic patients during the investigated time period.

### Foot ulcers in diabetic patients from the catchment area of KS amputated at KS

**Figure 4:**
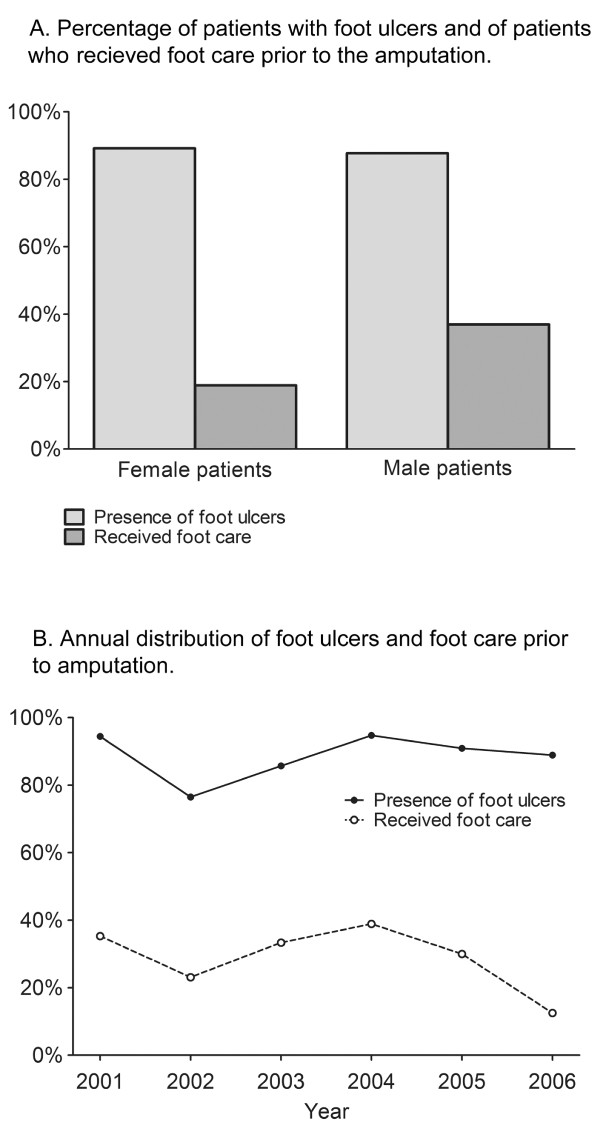
**Foot ulcers and foot care.** A. Percentage of patients with foot ulcers and of patients who received foot care prior to the amputation. B. Annual distribution of foot ulcers and foot care prior to amputation.

31 (30%) of the 102 diabetic patients from the catchment area of KS amputated at KS had been treated by the multidisciplinary foot team as either outpatients at the special foot clinic at the DEMD, or as inpatients at the same department with the diagnosis of foot ulcers (L98.4). The foot ulcer and foot care status of the 21 patients amputated at DS were not known, hence these patients could not be included in the analysis. Out of the 530 patients who were outpatients at the foot clinic, 68 (13%) were amputated during the time period. Of the 313 patients who were inpatients at the DEMD due to complicated foot ulcers, 52 (17%) underwent amputations. This number is higher than 31 since also patients from other catchment areas were treated at the foot clinic. The most severe non-healing chronic foot ulcers were referred from the primary care and constituted 25-33% of all patients with foot ulcers. In total, 90 (88%) of the 102 amputations were performed as a consequence of foot ulcers (Table 3). There was no significant difference between the female (89%) and male (88%) patients in the presence of foot ulcers prior to amputation (Figure 4A). However, there was a trend that more male patients had been referred to the foot clinic of the DEMD for foot ulcer treatment compared to the female patients (*p *= 0.07). Only seven (19%) of the female patients who were amputated had been treated as either in- or outpatients at the DEMD during the time period, compared to 24 (37%) of the male patients. 68% of the patients who had been inpatients at the DEMD due to foot ulcers (L98.4) were males (data not shown). Figure 4B shows the annual distribution of amputations performed on limbs affected by foot ulcers, compared to the distribution of patients receiving foot care. Statistical analyses did not reveal any linear trends in the presence of foot ulcers or foot care status over time.

### Comorbidities

**Figure 5:**
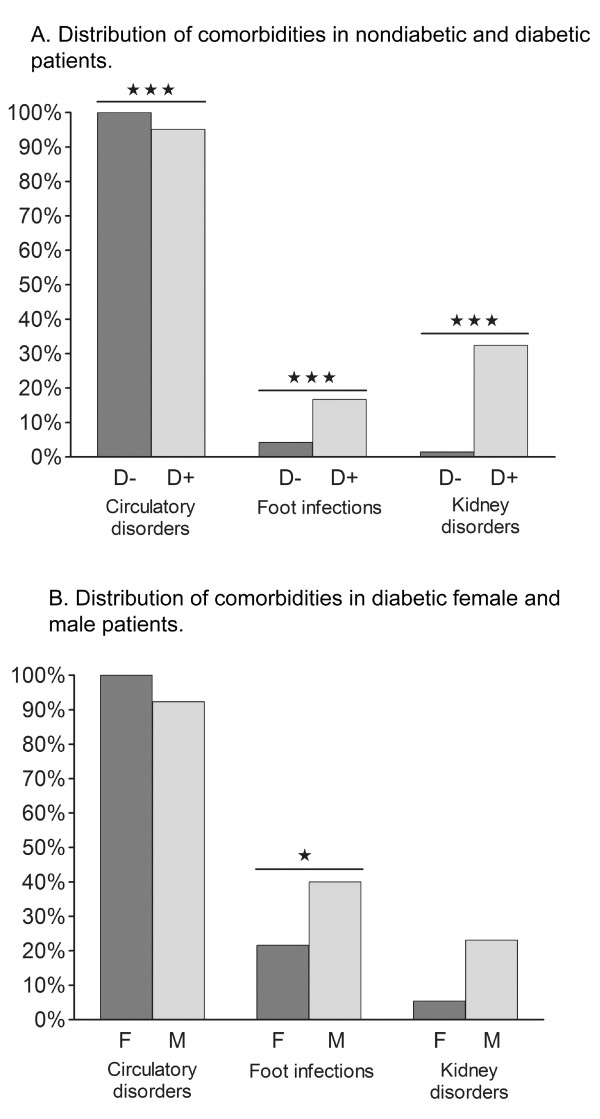
**Comorbidities.** A. Distribution of comorbidities in nondiabetic and diabetic patients. Unpaired *t *test: ***p < 0.001 D- *vs*. D+. B. Distribution of comorbidities in diabetic female and male patients. Unpaired *t *test: *p < 0.05 F *vs*. M.

There were primarily two different main diagnoses registered by the orthopaedic surgeons: insulin-dependent diabetes mellitus with peripheral circulatory complications (E10.5) and/or atherosclerosis of arteries of extremities with gangrene (I70.2). The DRG registration for diagnoses was not changed during the study period. Figures 5A and 5B show the accumulated distribution of additional diagnoses associated with an increased risk of LEA: circulatory disorders, foot infections, and kidney disorders. Diabetic patients suffered from more kidney disorders (*p *< 0.0001) and more foot infections (*p *< 0.0001) compared to nondiabetic patients amputated at KS (see Table 2 and Figure 5A). All nondiabetic patients suffered from vascular disorders, as this was a diagnosis criterion used for selecting the control group, whereas 86% of the diabetic patients were affected (*p *< 0.001). In the diabetic group, 17 (17%) patients suffered from severe foot infections, a condition more common in male patients (*p *< 0.05) (Figure 5B) and 33 (32%) patients suffered from kidney disorders, which tended to be more common in male patients (*p *= 0.08). 97 (95%) of the 102 diabetic patients suffered from circulatory disorders, including 88 (86%) patients with PVD. There was a trend of more PVD in female patients with diabetes (*p *= 0.08).

## Discussion

The number of major amputations decreased roughly by 60% in patients from the catchment area of KS during the investigated time period between 2001 and 2006. There was a tendency of a decrease in the total number of amputations, whereas the number of minor amputations remained stable, suggesting that diabetic patients underwent fewer and less disabling amputations at the end of the study period. Reductions in the total and major amputation rates are consistent with recent studies of trends in the amputation rate [17-19] and indicate an improved effectiveness in the diabetes care, possibly due to multidisciplinary actions.

The patients amputated in this study were predominantly male, a finding which is consistent with previous studies [20-22]. Male patients also underwent more re- and double amputations. The mechanisms underlying the increased rate of LEA in male patients is not known, but could be related to several factors, including previous smoking habits and a larger physical stress on the feet caused by increased height and body weight [23]. Another possible cause of the gender differences observed here is adherence, e.g. to which extent the off-loading advices were followed by the patients. Although little studied, treatment adherence is believed to have a substantial impact on the treatment outcome of the diabetic foot, and apart from identifying biochemical risk factors there is also a need to reveal individual personality traits that may pose an increased risk to foot complications and subsequent LEA.

An important aspect is that females may have more efficient wound healing due to the wound healing properties of estrogen receptor beta [24,25], whereas androgens are implicated to be detrimental to wound healing [26,27]. This correlates well to previous findings indicating that male sex is a risk factor for impaired wound healing [28], and is in line with males being more commonly affected by foot ulcers in western countries [29,30]. However, in Sweden type 2 diabetes is also more common in men overall [15], whereas that is not the case in adults with type 1 diabetes.

No sex-dependent age difference was seen in nondiabetic patients. However, female patients with diabetes were significantly older when amputated compared to male diabetic patients. The mean age of female diabetic patients in Sweden is 2 years higher (63.9 +/- 12.6) than in male diabetic patients (61.8 +/- 11.9) [31]. However, even though this difference is significant, it is probably not sufficient to explain the age difference of more than 10 years seen between the amputated diabetic female and male patients in this study. Apart from age *per se*, increased diabetes duration is an important risk factor for LEA [29,30]. Type 1 diabetes has an early onset and is associated with a high risk of LEA [32]; by the age of 65 years the cumulative risk for LEA was 10% in women and 20.7% in men with type1 diabetes [12]. The diabetes type and duration in our study cohort was unknown. However, in view of other Swedish studies there is a high probability that many patients in our cohort suffered from type 1 diabetes and that males were more commonly affected. This would mean that many of the male subjects had an earlier onset of diabetes, and hence an increased diabetes duration when undergoing LEA. It is important to keep in mind that many patients with type 1 diabetes have had a diabetes duration of 50 years or more at the age of 65 years. Moreover, it should be noted that the mean and median ages in the amputated females were remarkably high (81.5 and 84 years, respectively). In 2006, the mean life expectancy of the Swedish population was 82.8 years for females and 78.4 years for males [33], indicating that > 50% of the female diabetic patients were older than the mean expected maximum age when amputated. Information on blood pressure or blood lipid levels was not available in this patient material, but data from NDR, the Swedish national diabetes registry, revealed that females have less well controlled lipids and blood pressure (OR for male *vs*. female: blood pressure < = 130/80 mmHg = 1.05; S-cholesterol < 4.5 mmol/L = 1.8; LDL-cholesterol mmol/L < 2.5 = 1.28; S-triglycerides < 1.7 = 1.08) [34]. This information would rather indicate an increased risk of LEA in female patients suffering from diabetes, as PVD is a risk factor of amputations [2,29,30]. Nevertheless, female and male patients with diabetes were equally affected by CVD, but the overall mean age differed between the sexes, with female diabetic patients being significantly older. Consistent with previous data, this suggests that females can be protected from development of CVD decades after menopause [35].

Diabetic nephropathy is a major diabetic complication and a leading cause of end-stage renal disease [36]. Renal disorders are associated with an increased risk of neuropathy and PVD [8] and an increased risk of developing foot complications, including foot ulcers, infections, gangrene and LEA [19,30,37-39]. In this study, we found that diabetic patients who were amputated were significantly more affected by kidney disorders compared to nondiabetic patients. Furthermore, there was a trend towards more kidney disorders in male diabetic patients, which correlates well with the increased risk of LEA observed in this group. It has previously been suggested that the rate of foot complications in diabetic patients with end-stage renal disease and peritoneal dialysis might be reduced by a multidisciplinary approach and the early intervention of a chiropodist [40].

There was no gender difference in the presence of foot ulcers prior to amputation in patients with diabetes, indicating that once a foot ulcer is present the risk of LEA is similar in females and males. Nevertheless, delayed wound healing in male patients suggests increased vulnerability to ulcer infections, indicating that this could be a valid contributing factor to the significantly higher number of male patients affected by foot infections in our study. Notably, although female and male patients had the same prevalence of foot ulcers, only one fifth of the female patients who were amputated had been treated at the foot clinic at the DEMD at KS during the study period, compared to more than one third of the male patients. In a survey of the prevalence of diabetic foot ulcers in Stockholm county (1999, not published) around 3% of the diabetic population in primary care were treated for a foot ulcer at a given time. Only one third of all patients with foot ulcers were referred from the primary care. It is possible that foot ulcers in the older female patients were diagnosed as being primarily of vascular and not of diabetic origin, and therefore were not referred to the DEMD, whereas the men, who were approximately 10 years younger, were diagnosed with neuro-ischaemic diabetic foot ulcers and thus referred directly to the DEMD. As mentioned previously, there was no sex difference in the prevalence of diagnosed PVD, as opposed to what would be expected if the female patients had more ischaemic foot ulcers. Ischaemic foot ulcers are correlated to a more acute disease course and commonly require acute amputations [41], hence, these patients may not have the chance to receive preventive foot care in time. There is also a possibility that the female patients did more self-care, which to some degree could prevent complicated ulceration [42]. However, the large multi-centre American TRIAD study on a cohort of 8763 diabetic patients found no gender difference in the health behavior and amount of self-care in diabetic patients [43]. These conflicting findings might be related to ethnic differences, thus, gender and its impact of protective self-care remains speculative.

Neuropathy is a major risk factor for foot ulcers. In a recent study, Kärvestedt and colleagues [15] investigated diabetic subjects from a geographically defined population from Sundbyberg, a region which is part of the catchment area of KS, where 3.5% of the population between 40 and 70 suffered from diabetes. In this population 90% of the diabetic subjects had type 2 diabetes, and 34% of the patients with type 2 diabetes were affected by peripheral sensory neuropathy. This and other studies [44-47] suggest that many patients in our cohort were affected by neuropathy. Autonomic neuropathy increases the risk of ulceration by causing anhidrosis and oedema of the foot [48], and peripheral sensory polyneuropathy reduce the protective sensation of the distal limbs [49]. It has been proposed that peripheral neuropathy is associated with altered vascular function and endoneural hypoxia caused by PVD [50,51]. Furthermore, the nervous system interacts with the immune system [52,53], suggesting that the local immunity of diabetic patients with neuropathy may be altered. In line with this, diabetic patients are at a greater risk of severe infections during vascular surgery [54]. Consistently, we here report that the prevalence of infections of the foot prior to amputation was higher in diabetic compared to nondiabetic patients, notably with diabetic men being more commonly affected compared to diabetic women.

Our study has several strengths. Since all patients who were amputated at KS had their case reports read through, it is unlikely that any diabetic patients were missed in the cohort. This procedure also provided reliable identification of additional diagnoses and foot ulcers compared to interviews or self-reports. A limitation of our study was that the additional diagnoses and foot status of patients amputated at DS were not known, thus, these patients could not be used for analyses of comorbidities or foot status. However, as the patient group amputated at DS was referred due to geographic location only, it was expected to be similar to the group amputated at KS in respect of clinical characteristics. The diabetes type, duration, and the neuropathy status of the diabetic patients were unknown. However, since the Swedish diabetic population is well studied, it is possible to make valid assumptions based on previous studies. Another limitation was the coinciding change from paper case reports to a digital case report system before and during the beginning of the investigated time period. This made parts of the older case reports difficult to obtain, and it is possible that some amputations performed during the beginning of the study period went undetected. At KS, there were five basic levels of amputations registered (NHQ16, NHQ14, NGQ19, NGQ09, NFQ19), and to ensure that all registered amputations were correctly labeled, all codes must be compared to the surgeon's notes. This was not done in this study, which is a limitation. However, the recording of amputation codes has been computer based since many years, and is checked by a secretary as it is the base for the internal economic compensation system. This should limit the risk of erroneous reporting.

According to the consensus guidelines of foot care, better treatment outcomes are expected if all patients with problems related to the diabetic foot are treated by multidisciplinary treatment teams. However, our study revealed that despite the establishment of consensus guidelines, many patients who subsequently underwent amputations at KS had not received any multidisciplinary treatment through the DEMD. Nevertheless, the reduced major amputation rate indicates knowledge and awareness of the consensus guidelines on treatment of the diabetic foot. It cannot be excluded that amputation rates could be further reduced if all patients had been referred to the multidisciplinary foot team early, when the first symptoms of diabetes-related foot complications appeared. More efforts can be put into ensuring that patients being at risk of LEA will receive preventive foot care in time, and specialist treatment as soon as a foot ulcer is noticed, preferably within two weeks. These actions would possibly lead to further prevention of amputations in diabetic patients.

## Conclusions

• The rate of major amputations in diabetic patients decreased with approximately 60% between 2001 and 2006.

• Diabetic patients who were amputated had a higher prevalence of common comorbidities, including foot infections and kidney disorders, compared to amputated nondiabetic patients. Diabetic patients also underwent more re- and double amputations.

• Male diabetic patients were 10 years younger at the event of amputation, underwent a larger number of re-amputations and had a higher prevalence of infected foot ulcers compared to female diabetic patients.

• Only 30% of the patients had been in contact with the multidisciplinary foot team at the Department of Endocrinology, Metabolism and Diabetes before amputation.

• The amputation rate could possibly be further reduced if all patients at risk of LEA were referred to the multidisciplinary foot team in order to get specialist treatment.

## Abbreviations

CVD: Cardiovascular Disorders; DEMD: Department of Endocrinology: Metabolism and Diabetes; DS: Danderyd University Hospital; KS: Karolinska University Hospital: Solna; LEA: Lower Extremity Amputations; Ns: Non significant; PVD: Peripheral Vascular Disorders; OR: Odds Ratio.

## Competing interests

The authors declare that they have no competing interests.

## Authors' contributions

AA collected and analyzed the data, interpreted the results and wrote the manuscript. CW, BS and KB contributed with patient data. MA collected data from NDR, analyzed the data and revised the manuscript. KB designed the study, interpreted the results and revised the manuscript. All authors read and approved the final manuscript.
